# Wet‐dry cycles control the emissions and sources of greenhouse gases in agricultural soil: An incubation study

**DOI:** 10.1002/jeq2.70062

**Published:** 2025-07-20

**Authors:** Matt Ball, Guillermo Hernandez‐Ramirez

**Affiliations:** ^1^ Department of Renewable Resources Faculty of Agricultural, Life & Environmental Sciences University of Alberta Edmonton Canada

## Abstract

This study examines the impact of wet‐drying cycles and nitrogen (N) fertilization on soil greenhouse gas fluxes, specifically nitrous oxide (N_2_O) and carbon dioxide (CO_2_). Nine treatments were tested, combining three soil moisture regimes (55% constant, 55%–30% cycle, and 80%–55% cycle) with three N addition rates (0, 100, and 150 kg N ha^−1^) using ^15^N‐labeled urea. Soil samples from a potato (*Solanum tuberosum*) field in Lethbridge, Alberta, were incubated for 28 days under controlled conditions. Wet‐drying cycles involved initially wetting the soil to the upper threshold (55% or 80% WFPS) and allowing it to dry to the lower threshold (30% or 55% WFPS), followed by rewetting to restore upper moisture levels. N_2_O and CO_2_ fluxes were measured regularly using a recirculation chamber system to quantify gas emissions and determine N_2_O sources. Soil moisture significantly increased N_2_O and CO_2_ production (*p* < 0.001), with the highest emissions under wet conditions (80%–55% WFPS cycle), moderate production at 55% WFPS, and the lowest under dry conditions (30%–55% WFPS cycle). Compared to constant 55% WFPS, N_2_O and CO_2_ production were 33% and 403% higher, respectively, under wet conditions and 28% and 3% lower under dry conditions. Rewetting events triggered temporary increases in gas emissions due to enhanced microbial activity. Urea addition caused a stronger priming effect on N_2_O production under wet conditions, with urea‐derived N_2_O more prominent in wetter soils.

## Core Ideas

- Rewetting events triggered pronounced temporary increases in N_2_O and CO_2_ production.
- Pre‐existing soil organic matter nitrogen (SOM‐N) was the dominant source of N_2_O.
- Urea‐derived N_2_O disproportionally increased with wetter conditions and higher N addition.
- Priming effects on N_2_O were minimal, though priming was enhanced by increasing soil moisture.

**Abbreviations**
ANOVA: analysis of variance
EC: electrical conductivity
PE: priming effect
SOM: soil organic matter
WFPS: water‐filled pore space

## 1 INTRODUCTION

Investigating greenhouse gas production from fertilized soil under wet‐dry cycles holds major importance in the fields of environmental science and climate change mitigation. This interplay between soil moisture fluctuations and N fertilization practices can profoundly influence the release of greenhouse gases, such as nitrous oxide (N_2_O) and carbon dioxide (CO_2_), into the atmosphere. Nitrification and denitrification processes have been observed to co‐occur during wet‐dry cycling, causing the generation of large pulses of greenhouse gas production (Pinto et al., 2022).

Understanding the implications of this dynamic is valuable for several reasons. Agriculture is a major contributor to global greenhouse gas emissions, estimated at one third of anthropogenic emissions (Gilbert, 2012), with soil management practices accounting for a significant portion. Therefore, by better understanding how wet‐dry cycles influence greenhouse gas production, targeted strategies can be developed to minimize the environmental footprint of agricultural activities. Additionally, wet‐dry cycles are becoming more irregular and pronounced under climate change, due to increased instances of droughts and heavy rainfall (Clarke et al., 2022). This therefore increases the frequency of wet‐dry cycling events in soil globally, while also heightening total emissions from such cycling and their significance.

Climatic context plays a key role in shaping soil responses to these wet‐dry cycling. Temperate, Mediterranean, and tropical systems exhibit contrasting microbial adaptations to moisture stress and rewetting, with these biome‐specific sensitivities being crucial for fully understanding soil responses to moisture dynamics (Barnard et al., 2020; Barrat et al., 2021). These contrasting responses are driven by geographic differences in the intensity and frequency of wet‐dry transitions, referred to as wet‐dry intensity gradients. These gradients can variably affect microbial processes and gaseous emissions depending on the duration and magnitude of drying and rewetting. Stronger drying phases followed by intense rewetting often stimulate microbial turnover, enhance substrate availability, and elevate greenhouse gas fluxes (Barnard et al., 2020; Barrat et al., 2021). Furthermore, the historical moisture status of a soil significantly influences its N₂O response, with drought‐affected soils exhibiting higher emissions upon rewetting compared to consistently moist soils (Barrat et al., 2021).

While prior studies have investigated the relationship between wet‐dry cycling and greenhouse gas production (i.e., Harrison‐Kirk et al. [2013] and Pinto et al. [2022]), the number of studies that have considered wet‐dry cycling within an agricultural context remains limited. Additionally, at present, a key aspect of N_2_O emissions that remains underexplored—in relation to wet‐dry cycling—is the contribution of priming effects (PE) to overall production and the relative contributions of cumulative N_2_O emissions from urea addition and soil organic matter nitrogen (SOM‐N). The sources of N_2_O emissions in agricultural soil are primarily linked to denitrification and nitrification, which are driven by the availability of reactive nitrogen (N) forms like ammonium (NH₄⁺) and nitrate (NO₃⁻) (M. U. Hassan et al., 2022). These processes occur in different soil pools, with urea fertilization contributing directly to the available N pool, while soil organic matter (SOM) serves as a reservoir of N that is mineralized by microbes over time (Chien & Krumins, 2023). When urea is applied, it can stimulate microbial activity, not only by providing an immediate source of NH₄⁺ but also by “priming” the decomposition of SOM, thus releasing additional inorganic N (Daly et al., 2024; Kuzyakov et al., 2000). This priming can lead to enhanced N_2_O emissions from both newly available urea‐derived N and from SOM‐N, which may otherwise have remained less active (Daly & Hernandez‐Ramirez, 2020; Roman‐Perez & Hernandez‐Ramirez, 2021; Thilakarathna & Hernandez‐Ramirez, 2021b). Therefore, gaining a deeper understanding of the interconnection between greenhouse gas production, N fertilization, and wet‐dry cycling offers a pathway toward more informed agricultural practices and greater climate change mitigation.

Insights into the control of wet‐dry cycling on greenhouse gas production from potato (*Solanum tuberosum*) field soil, specifically, are uniquely valuable. Commercial potato production commonly involves irrigation to maximize potato yields, quality, and overall profitability (Akkamis & Caliskan, 2023; Alva et al., 2012). This is primarily due to the specific drought sensitivity of potatoes caused by their shallow root system (Joshi et al., 2016). Additionally, N₂O emissions from potato production systems have been shown to decrease as a greater proportion of cumulative water input comes from irrigation rather than rainwater (Ball & Hernandez‐Ramirez, 2025). This reduction is attributed to the greater control of soil moisture in irrigated systems compared to rainfed systems, which substantially lowers the likelihood of excess soil moisture (Ball & Hernandez‐Ramirez, 2025; Schoengold & Zilberman, 2007). However, this observation has yet to be fully elucidated—in relation to the relative effects of dry‐fluctuating and wet‐fluctuating conditions compared to short‐term moisture fluctuations on greenhouse gas production. Therefore, the objective of this study is to examine how soil moisture—including wetting and drying events—alongside N fertilization influence N_2_O and CO_2_ production, while also assessing the sources and priming effect on N_2_O production.

## 2 MATERIALS AND METHODS

A laboratory incubation study was conducted to investigate the impact of wet‐dry cycling, through differing water‐filled pore space percentages and wetting/drying rates, alongside N fertilization on greenhouse gas fluxes from soil.

### 2.1 Soil collection and field characteristics

A composite soil sample (0‐ to ‐15‐cm depth) was prepared by combining soil cores collected using a hand corer from multiple locations within a potato field at the Irrigation Demo Farm, at Lethbridge Polytechnic (49° 47′ 59.2548″ N, 112° 45′ 20.7612″ W) at the beginning of the growing season in May. The field follows an annual crop rotation of barley (*Hordeum vulgare*), sugar beet (*Beta vulgaris*), wheat (*Triticum aestivum*), and potato. The soil is classified as Dark Brown Chernozem with a sandy clay loam texture. The soil had a pH of 7.46 (±0.05) and an EC (electrical conductivity) of 145 (±32) dS m^−1^, with C and N of 18.8 ± 7.3 and 1.53 ± 0.65 mg kg, respectively.

### 2.2 Incubation design

After sampling, the soil samples were mixed and sieved with an 8‐mm mesh prior to a pre‐incubation period. After mixing, samples of the mixed soil were collected and dried in an oven for 24 h (60°C) to measure the moisture content. Nine specific treatments were investigated (with four replicates each), consisting of three separate soil moisture conditions, alongside three rates of N addition (Table 1). The soils were incubated in circular plastic containers measuring 7 cm in height with a 5‐cm inner diameter, each microcosm was packed with 131 g of soil (oven‐dry equivalent) to a bulk density of 1.0 g cm^−3^, mirroring soil conditions in the field. The N input (fertilizer) to the microcosms was ^15^N labeled urea (at 5 atom%) incorporated into the containers following the day 1 measurements, to simulate fertilizer additions. The rates of ^15^N labeled urea were 0, 10.87, and 16.30 mg (equivalent to rates of 0, 100, and 150 kg N ha^−1^). For conducting measurements, a chamber with a headspace of 0.5 L was established enclosing an incubation container in a Mason jar.

**Core Ideas**

- Rewetting events triggered pronounced temporary increases in N_2_O and CO_2_ production.
- Pre‐existing soil organic matter nitrogen (SOM‐N) was the dominant source of N_2_O.
- Urea‐derived N_2_O disproportionally increased with wetter conditions and higher N addition.
- Priming effects on N_2_O were minimal, though priming was enhanced by increasing soil moisture.

**TABLE 1:** List of experimental treatments detailing addition rates of urea and water‐filled pore space (WFPS) regimes.

Rate of urea addition (kg N ha^−1^)	Rate of urea addition (mg urea per soil microcosm)	WFPS (%) regime
0	0	80–55
0	0	55–30
0	0	55
100	10.87	80–55
100	10.87	55–30
100	10.87	55
150	16.30	80–55
150	16.30	55–30
150	16.30	55

**FIGURE 1:**
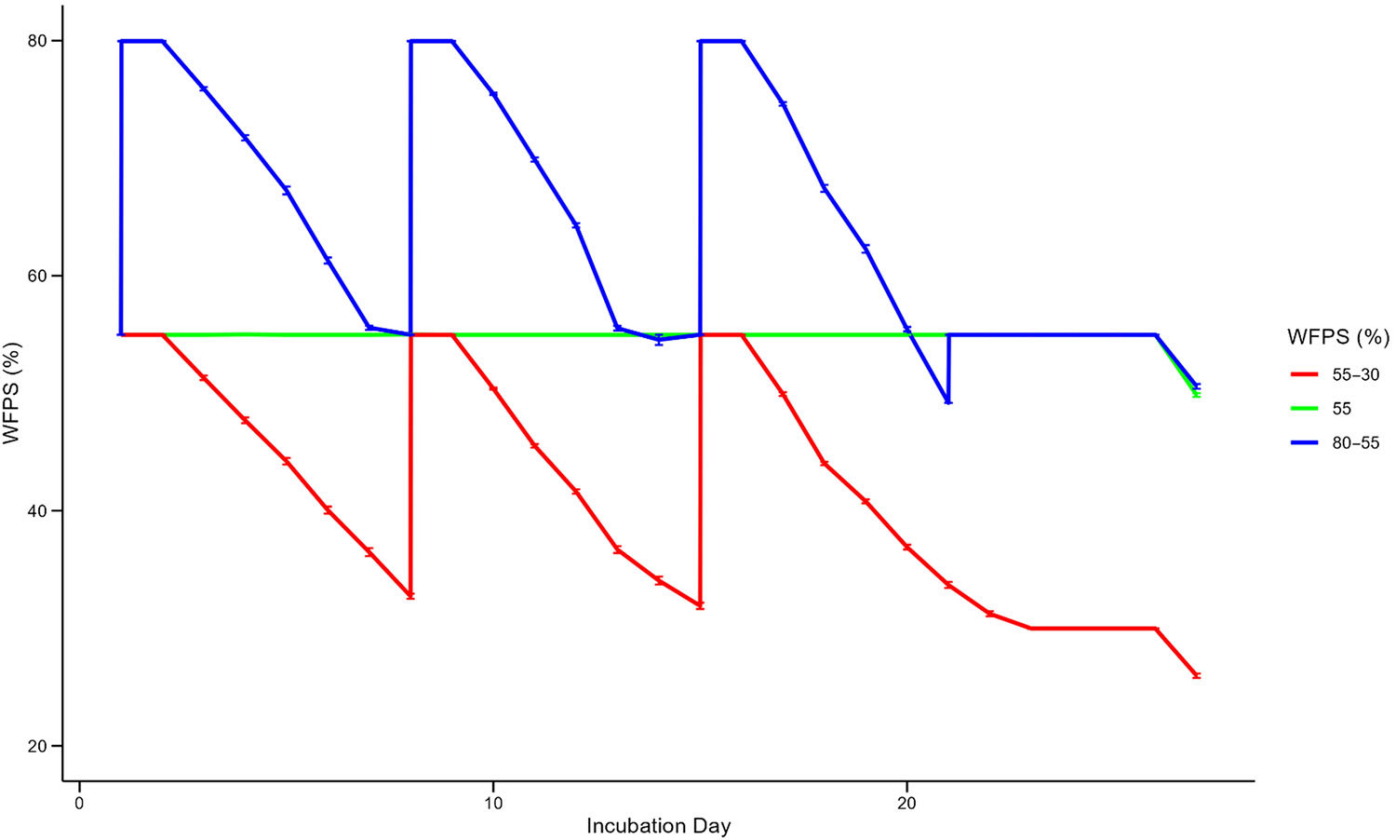
Daily soil water‐filled pore space (WFPS) (%) during the incubation by WFPS treatment. Error bars represent ± 1 SE (standard error) for each condition.

For the wet‐fluctuating and dry‐fluctuating soil conditions, the soil was wetted up to the upper water‐filled pore space % (WFPS) bound (80% or 55%), before letting it gradually dry to the lower bound (55% or 30%) and then rewetted again to restart the successive cycles. For the constant‐baseline conditions, soil WFPS was held constant at 55%—due to 55% being calculated as a typical WFPS within potato production (Snowdon et al., 2013). The lower and upper bounds of 30% and 80% are specifically chosen for being 25% greater and less than this typical WFPS, respectively (Figure 1).

Water‐filled pore space was calculated as:

(1) $$\mathrm{WFPS}\%=\frac{\left(\mathrm{GWC}\%\;\times 100\right)}{f},$$

where WFPS is water‐filled pore space (%), GWC% is gravimetric water content (%) and *f* is the soil porosity (cm^3^ cm^−3^) (Linn & Doran, 1984).

Soil porosity was calculated as:

(2) $$f=1-\left(\mathrm{BD}÷\mathrm{PD}\right)\times 100,$$

where BD is soil dry bulk density (g g^−1^) and PD is soil particle density assumed as 2.65 g cm^−3^.

Gravimetric water content was calculated as:

(3) $$\mathrm{GWC}\%=\frac{M_{W}}{\mathrm{BD}\times \mathrm{VT}}\times 100,$$

where GWC% is gravimetric water content (%), *M_W_* is mass of water (g), BD is soil dry bulk density, and V_T_ is total volume (cm^3^).

Soil dry bulk density was calculated as:

(4) $$\mathrm{BD}=M_{D}÷V_{T},$$

where BD is soil dry bulk density, *M_D_* is mass of dry soil (g), and *V_T_* is total volume (cm^3^).

And mass of water was calculated as:

(5) $$M_{w}=M_{T}\;-M_{D},$$

where *M_W_* is mass of water (g), *M_T_* is total mass of soil (g), and *M_D_* is mass of dry soil (g).

A pre‐incubation period lasted 3 days prior to the incubation, where each soil container was kept at a constant temperature and WFPS (55%) to equilibrate soil microbial activity prior to measurements beginning. The incubation ran until three wet‐dry cycles had been completed by each treatment. The containers were left open and maintained at room temperature (22°C; HOBO temp logger) on the benchtop in the laboratory throughout the entire incubation period, with soil WFPS monitored daily by recording weight changes using a precision balance (± 0.1 g). When adjustments were needed, deionized water at room temperature was added dropwise around the container using a syringe to replenish the target WFPS. N_2_O and CO_2_ concentration changes with time for each soil microcosm were measured on incubation days 1, 2, 3, 4, 6, 9, 10, 11, 13, 16, 17, 18, 20, 23, 25, and 27.

### 2.3 Greenhouse gas production measurements

A non‐steady‐state chamber system enabled simultaneous measurements of CO_2_ and N_2_O alongside the overall ^15^N‐N_2_O value. These measurements were conducted using a cavity ring‐down laser spectroscope (G2508; Picarro Instruments) for CO_2_ and a quantum cascade laser spectroscope (QC‐TILDAS; Aerodyne Research Inc.) for N_2_O connected through recirculation to a chamber where soil microcosms were positioned for measurements. Temperature and pressure within the chamber system were recorded during flux measurements, with the sample flow rate through the chamber headspace held constant at 2.1 standard L min^−1^.

The chamber systems were closed for a total of 3 min to gather continuous greenhouse gas measurements. A 30‐s interval between chamber system measurements was utilized to allow for a return to ambient concentrations and isotopic compositions before the next soil microcosm was measured.

N_2_O and CO_2_ production rates were determined through linear regression of the concentration increase in the chamber headspace over time, adjusting for chamber volume, soil mass, pressure, and temperature. The applied modified ideal gas law was as follows (Pennock et al., 2010; Yates et al., 2006):

(6) $$\mathrm{Production}\;\mathrm{rate}=\frac{P\;\times \;\mathrm{slope}\;\times \;W\;\times \;3600\;\times \;24\;\times \;V}{R\;\times \;T\;\times \;\mathrm{mass}},$$

where production rate is µg N_2_O‐N kg soil^−1^ day^−1^, or mg CO_2_‐C kg soil^−1^ day^−1^; *P* denotes the pressure in the chamber headspace (atm); slope is the linear regression coefficient during the 3 min when the chamber was enclosed (nL L^−1^ s^−1^); *W* is the weight of N (28 g mol^−1^) or C (12 g mol^−1^) within a mole of N_2_O or CO_2_; *V* is the volume of the chamber headspace (L); *R* is the universal gas constant (atm nL K^−1^ µmol^−1^); *T* is temperature in the chamber headspace (K); and mass is the dry soil mass (kg). Cumulative CO_2_ and N_2_O productions for the duration of the incubation were calculated by multiplying the average gas production rate of two consecutive measurements by the time interval between measurements.

### 2.4 Nitrous oxide source and priming calculations

The utilization of ^15^N‐labeled urea enabled the differentiation between N_2_O derived from urea and SOM sources (Daly & Hernandez‐Ramirez, 2020; Thilakarathna & Hernandez‐Ramirez, 2021a). The SOM source was considered to consist of N mineralized from SOM during the experiment and the initial presence of NH_4_^+^–N and NO_3_^−^–N.

The Keeling plot method, as described by Chen et al. (2016), was utilized to analyze the isotopic composition of N_2_O, as well as the intramolecular distribution of ^15^N–N_2_O in soil samples. This method involves plotting inverse concentration values against isotopic composition to identify the signature of the source process as follows:

(7) $$\begin{array}{ccc} \delta^{15}\;X_{\mathrm{sample}} & = & x_{\mathrm{background}}\left(\delta^{15}X_{\mathrm{background}}-\delta^{15}X_{\mathrm{soil}}\right)\\ & & \left(\frac{1}{x_{\mathrm{sample}}}\right)+\delta^{15}X_{\mathrm{soil}}, \end{array}$$

where $\delta^{15}$ *X*_sample_, $\delta^{15}$ X_background_, and $\delta^{15}$ X_soil_ represent the isotope ratio of the measured sample, the background (ambient) air, and the soil for ^15^N–N_2_O, respectively. Meanwhile, *X*_background_ and *X*_sample_ refer to the concentrations of the target component in the ambient air and the total measured sample, respectively (Pataki et al., 2003).

Additionally, a two‐source mixing model, outlined by Lu et al. (2018), was employed to differentiate N_2_O derived from SOM versus exogenous N inputs of urea:

(8) $$X_{\mathrm{soil}}=X_{\mathrm{total}}\frac{\left(\delta^{15}X_{\mathrm{added}}-\delta^{15}X_{\mathrm{total}}\right)}{\left(\delta^{15}X_{\mathrm{added}}-\delta^{15}X_{\mathrm{soil}}\right)},$$

(9) $$X_{\mathrm{added}}=X_{\mathrm{total}}-X_{\mathrm{soil}},$$

where *X*_soil_ represents the microbial utilization of SOM‐N, while *X*_total_ is the total N_2_O emitted from the treatment. The $\;\delta^{15}$ *X*_added_, $\;\delta^{15}$ *X*_total_, and $\;\delta^{15}$ *X*_soil_ correspond to the δ^15^N values of N_2_O–N from urea, total N_2_O, and SOM‐N, respectively.

While PE were calculated according to Zang et al. (2016) by comparing N_2_O production in N‐amended soils and control soils:

(10) $$PE=\left[N_{2}O_{X\mathrm{soil},\;\;\mathrm{fertilized}}\right]-\left[N_{2}O_{X\mathrm{soil},\;\;\mathrm{control}}\right],$$

where [N_2_O*_X_*_soil, fertilized_] and [N_2_O*_X_*_soil, control_] indicate the N_2_O production from SOM‐N in the ^15^N fertilized and control soils, respectively.

### 2.5 Soil analyses

Soil δ^15^N analysis (continuous flow isotope ratio mass spectrometry), along with potassium chloride extractions analyzed by colorimetry to quantify NO₃⁻ and NH₄⁺ concentrations, was performed. Analysis was conducted on pre‐incubated soil to quantify initial levels (T0) using four pots set aside for destructive sampling. In addition, 12 more pots (four replicates of three different treatments: 0 kg ha^−1^ at constant 55% WFPS, 100 kg ha^−1^ at 55%–30% WFPS cycle, and 150 kg ha^−1^ at 80%–55% WFPS cycle) were set aside and destructively sampled after day 10. Finally, all 36 pots—measured for N₂O and CO₂ production throughout the incubation—were analyzed at the end of the incubation. Soil total C and total N analysis was performed using dry combustion. Soil urea‐N retention at the conclusion of the incubation experiment was calculated using soil δ^15^N and total N concentration data, along with standard mass balance calculations. Sampled soil pH and EC were also measured using corresponding laboratory probes (1:1 soil:water dilution).

### 2.6 Statistical analyses

Cumulative N₂O and CO₂ production, PE, N_2_O source allocations as well as soil urea‐N retention were assessed using a two‐way analysis of variance (ANOVA), with moisture regime and N fertilization rate as fixed effects. Available N (NH₄⁺ and NO₃⁻) concentrations were analyzed using a two‐way Welch's ANOVA due to violations of the assumption of homogeneity of variances. PE and available N concentrations were log‐transformed prior to analysis to correct for violations of the assumption of normality. Normality was assessed with Shapiro–Wilk tests, and homogeneity of variance was assessed with Levene tests. Post hoc comparisons were conducted using Tukey's honest significant difference (HSD) test to identify specific group differences when significant effects were found. All analyses were performed at an alpha critical value of 0.05.

## 3 RESULTS

### 3.1 N_2_O production

**FIGURE 2:**
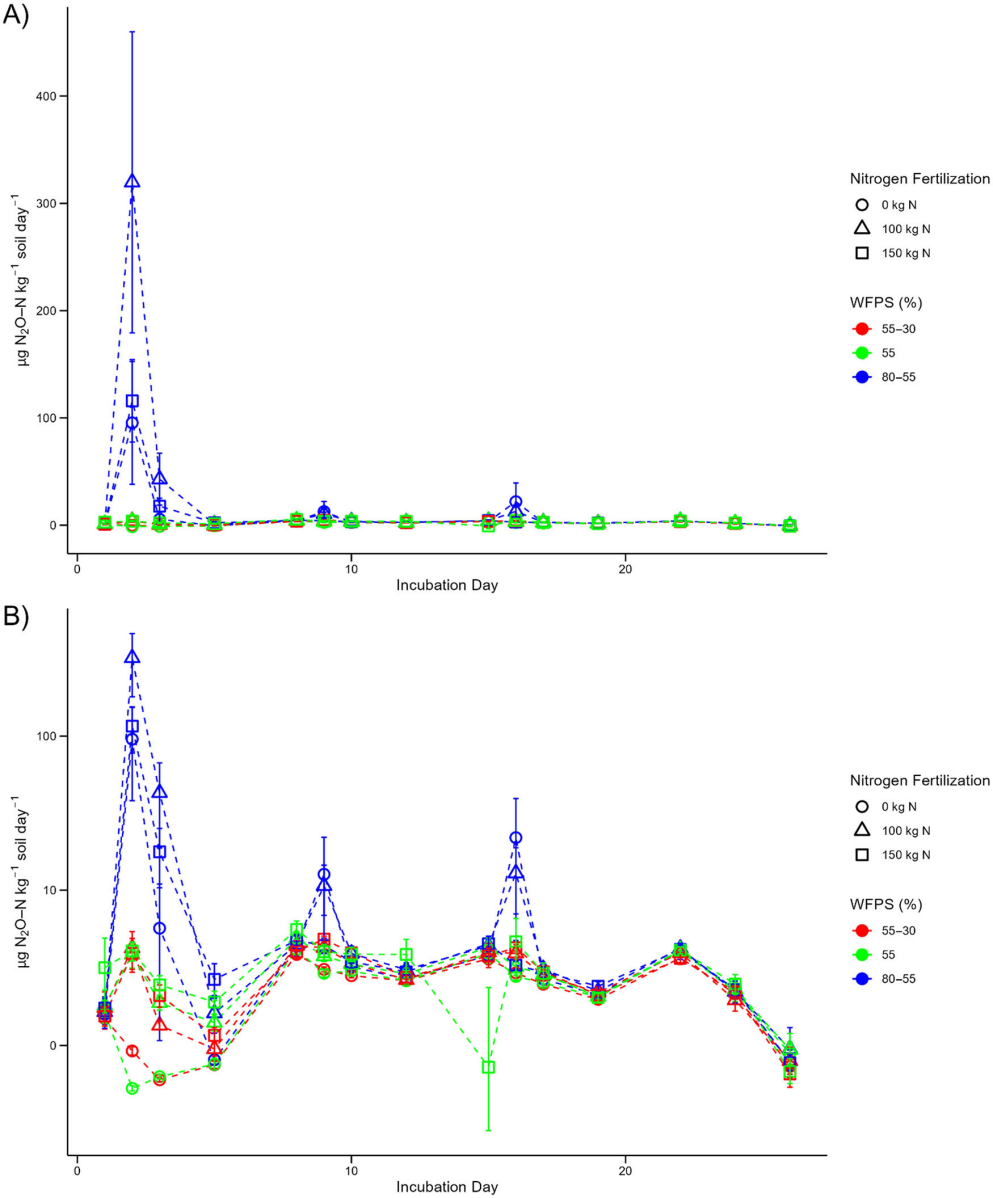
Daily N_2_O production (µg N_2_O–N kg^−1^ soil day^−1^) during the incubation by treatment: (A) Untransformed scaling and (B) Pseudo‐log scaling of the *Y*‐axis. Error bars represent ± 1 SE (standard error) for each treatment.

Overall, daily N_2_O production was largely constant under all the dry‐fluctuating and constant‐baseline moisture treatments (Figure 2). However, production was increased on day 8 onward, before decreasing from day 22 onward, under the dry‐fluctuating and constant‐baseline moisture treatments (Figure 2). In contrast, the wet‐fluctuating treatments exhibited much higher daily N_2_O production overall (Figure 2). On day 2 of the incubation, the wet‐fluctuating treatments had the highest recorded N_2_O production of the experiment (Figure 2). Additional increases in daily N_2_O production were observed on day 9 and 16, each being the first measurement after the soil was rewetted back to 80% WFPS (Figures 1 and 2).

Cumulative N_2_O production, across the incubation, was controlled in majority by the applied moisture regime, which was highly significant (*p* < 0.001) (Table 2). The pots that experienced a wet‐fluctuating moisture regime all produced significantly higher cumulative average rates of N_2_O, compared to the dry‐fluctuating and constant‐baseline moisture pots (*p* < 0.01) (Table 2). The control of N addition rate alone was found to be nonsignificant across the treatments (*p* > 0.05). However, through post hoc analysis, the average cumulative N_2_O production from wet‐fluctuating pots fertilized with 100 kg N ha^−1^ equivalent of ^15^N urea was identified as being significantly higher than wet‐fluctuating pots fertilized with 0 and 150 kg N ha^−1^, respectively (*p* < 0.05) (Table 2). No significant differences in cumulative N_2_O production between any of the dry‐fluctuating or constant‐baseline moisture treatments, irrespective of N fertilization rate, were identified (*p* > 0.05) (Table 2).

**TABLE 2:** Cumulative CO_2_, N_2_O, and N_2_O priming production, alongside the distribution of cumulative N_2_O production from soil organic matter nitrogen (SOM‐N) and urea.

Treatment	Mean cumulative CO_2_ production (mg CO_2_‐C kg^−1^ soil)	Mean cumulative N_2_O production (µg N_2_O–N kg^−1^ soil)	Mean cumulative N_2_O priming production (µg N_2_O–N kg^−1^ soil)	Proportion of cumulative N_2_O production from urea addition (%)	Proportion of cumulative N_2_O production from SOM‐N (%)
0 kg N ha^−1^ 80%–55% WFPS	13.36 ± 4.93ab	184.02 ± 139.06a	N/A	N/A	100
0 kg N ha^−1^ 55%–30% WFPS	7.44 ± 1.24b	44.06 ± 7.94b	N/A	N/A	100
0 kg N ha^−1^ 55% WFPS	11.76 ± 3.58b	37.07 ± 26.01b	N/A	N/A	100
100 kg N ha^−1^ 80%–55% WFPS	16.65 ± 2.51a	462.67 ± 294.92c	0.61 ± 0.02a	29.36 ± 10.10ab	70.64 ± 10.10
100 kg N ha^−1^ 55%–30% WFPS	7.45 ± 3.72b	59.51 ± 11.68b	0.01 ± 0.00b	7.8 ± 0.04c	92.2 ± 0.04
100 kg N ha^−1^ 55% WFPS	9.54 ± 1.09b	64.79 ± 15.79b	0.02 ± 0.00b	11.21 ± 1.01c	88.79 ± 1.01
150 kg N ha^−1^ 80%–55% WFPS	12.95 ± 2.08ab	207.74 ± 71.57a	0.56 ± 0.02a	32.38 ± 1.85a	67.62 ± 1.85
150 kg N ha^−1^ 55%–30% WFPS	8.43 ± 1.13b	61.50 ± 12.60b	0.01 ± 0.00b	14.35 ± 2.11b	85.65 ± 2.11
150 kg N ha^−1^ 55% WFPS	11.00 ± 2.10b	66.08 ± 13.98b	0.01 ± 0.00b	20.35 ± 8.60b	79.65 ± 8.60

*Note*: Values are the means ± 1 standard error across the four replicates. Letters indicate significant differences across treatments (*p* < 0.05).

Abbreviations: N/A, not available; WFPS, water‐filled pore space.

The allocations of N_2_O production, from urea addition and SOM‐N, are likewise primarily governed by the applied moisture regime (*p* < 0.001) (Table 2). Across all treatments, the proportion of N_2_O production from urea addition is observed to increase with increasing WFPS (Table 2). Across all rates of N fertilization, the wet‐fluctuating treatments consistently saw the highest proportions of N_2_O production from urea addition, which decreased under the constant‐baseline moisture treatments and was the lowest under the dry‐fluctuating treatments (Table 2). The effect of N fertilization itself was nonsignificant; however, the proportion of N_2_O production from urea addition significantly increased under 150 kg N ha^−1^ compared to 100 kg N ha^−1^ (*p* < 0.05) (Table 2). While the proportions of N_2_O for the two sources vary with WFPS and N fertilization, the majority of N_2_O production in all the pots was derived from SOM‐N (Table 2).

### 3.2 Priming effects of urea on N_2_O production

**FIGURE 3:**
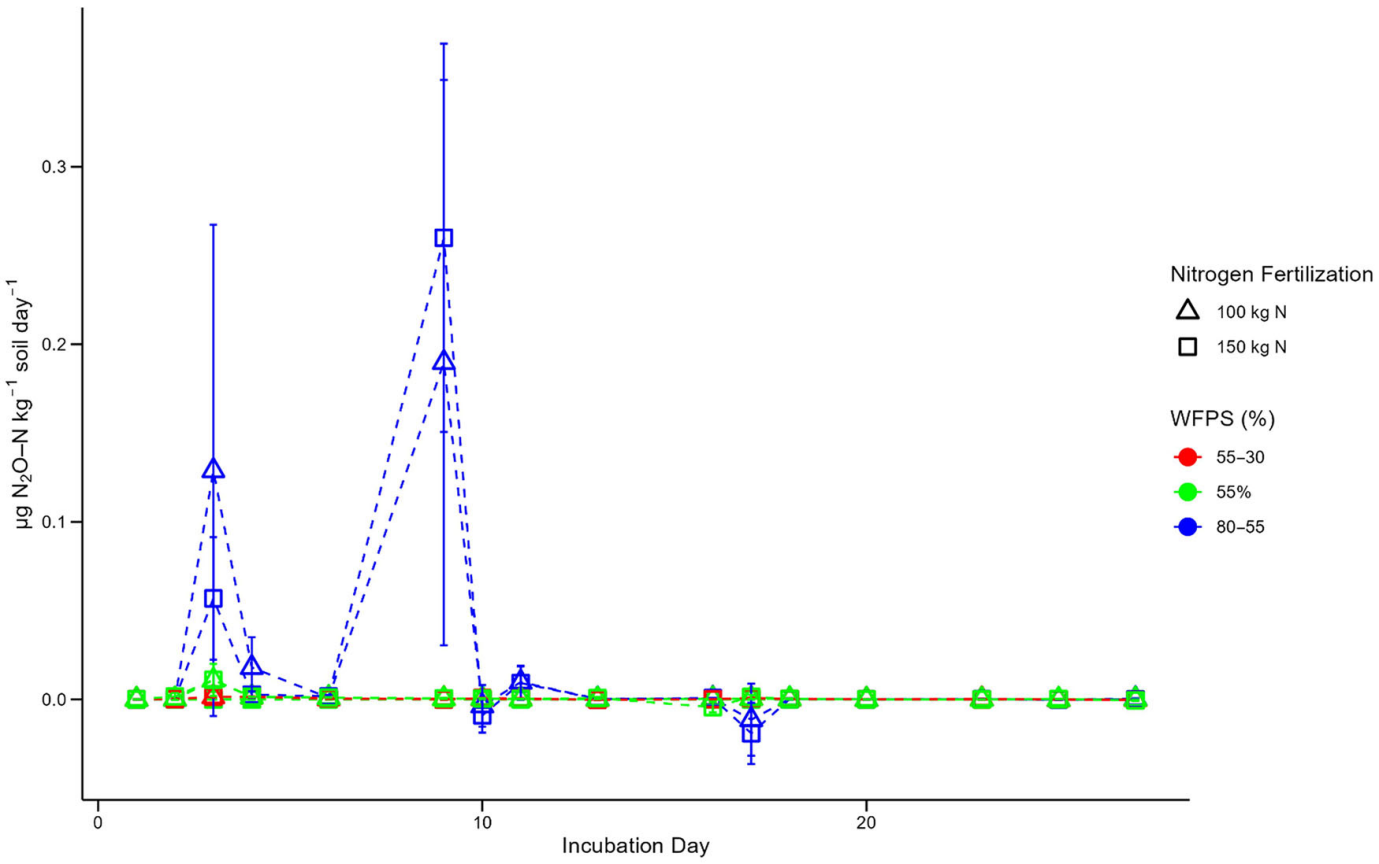
Daily priming of N_2_O production (µg N_2_O‐N kg^−1^ soil day^−1^) during the incubation across the N fertilized treatments. Error bars represent ± 1 SE (standard error) for each treatment.

PE on daily N_2_O production under the dry‐fluctuating and constant‐baseline moisture treatments were all near zero across the incubation (Figure 3, Table 2). Likewise, for the wet‐fluctuating treatments, PE were minimal—except for minor primed increases in N_2_O production on day 3 and 9 (Figure 3). Contrastingly, there was minimal variability within the moisture regimes based on N fertilization rate (Figure 3). Resultantly, the cumulative PE were small across the treatments; however, significant differences were identified between the moisture regimes (*p* < 0.05) (Table 2). The fluctuating wet moisture cycle (80%–55% WFPS) led to increased priming relative to both constant‐baseline (constant 55% WFPS) and fluctuating dry moisture cycle (55%–30% WFPS) treatments.

### 3.3 Available N in soils

**FIGURE 4:**
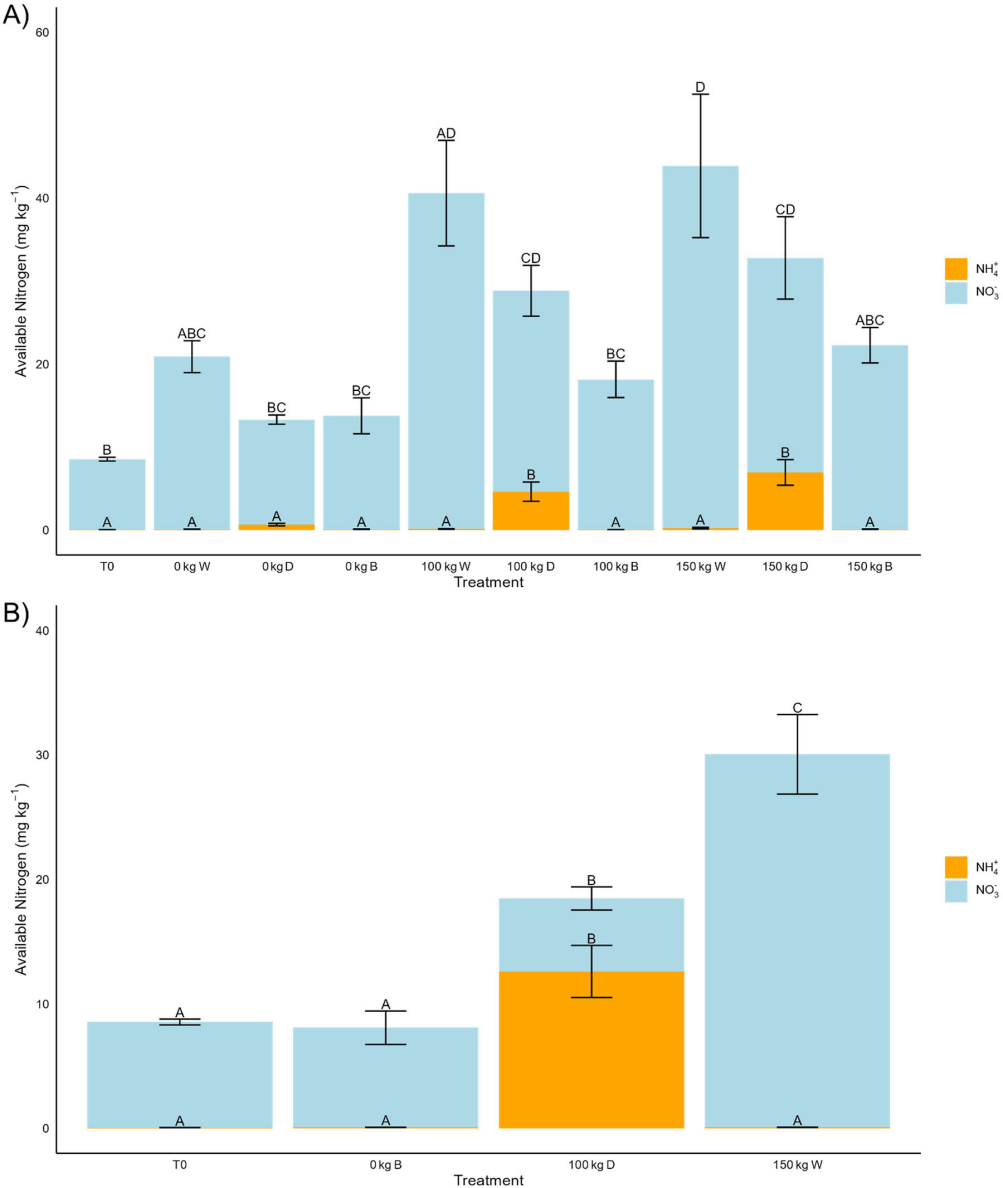
(A) Initial N concentrations (T0) of NO₃⁻ and NH₄⁺ and end of the incubation concentrations across treatments, and (B) concentrations after day 10 in three treatments. Letters indicate statistical differences between NO₃⁻ and NH₄⁺ across treatments (*p* < 0.05). Error bars represent ± 1 SE (standard error) for each treatment. 0, 100, and 150 kg refer to 0, 100, and 150 kg ha^−1^ of N fertilization, respectively. D, B, and W refer to dry‐fluctuating (30%–55% WFPS cycle), constant‐baseline (constant 55% WFPS), and wet‐fluctuating (80%–55% WFPS cycle) moisture regimes, respectively.

Soil NH₄⁺ and NO₃⁻concentrations were both affected significantly by the moisture regime and N fertilization rate of a given treatment. For NH₄⁺ concentrations, moisture was a highly significant control (*p* < 0.001) alongside N fertilization rate (*p* < 0.01). Likewise, for NO₃⁻ concentrations, moisture and N fertilization rate were both highly significant controls (*p* < 0.001). At the end of the incubation, soil NO₃⁻ concentrations increased across all treatments compared to the initial concentrations, with NO₃⁻ concentrations being much higher than NH₄⁺ concentrations in each case (Figure 4A). In the dry‐fluctuating treatments (55%–30% WFPS cycle), final NH₄⁺ concentrations were markedly higher than in other treatments (Figure 4A). Among these, the two treatments fertilized with urea had significantly higher NH₄⁺ concentrations than all others (Figure 4A). Conversely, the wet‐fluctuating treatments (80%–55% WFPS cycle) recorded the highest final NO₃⁻ concentrations for each concentration of N fertilization (0, 100, and 150 kg N ha^−1^) (Figure 4A). The NH₄⁺ and NO₃⁻ concentrations taken after 10 days follow the same trends observed with the final concentrations. The dry‐fluctuating treatment had the highest concentration of NH₄⁺ across the treatments, while the wet‐fluctuating treatment had the highest NO₃⁻ concentration (Figure 4B). Overall available N again increased with N fertilization rate, being the lowest with 0 kg N ha^−1^ and the highest with 150 kg N ha^−1^ (Figure 4B).

### 3.4 Urea‐N retention

No significant differences (*p* > 0.05) in soil urea‐N retention were observed among the treatments at the conclusion of the experiment. The control soils had an average δ^15^N of 9.65 ± 0.47 ‰ (standard error). The soils that received ^15^N‐labeled urea had an average δ^15^N of 10.28 ± 0.70 ‰.

### 3.5 CO_2_ production

**FIGURE 5:**
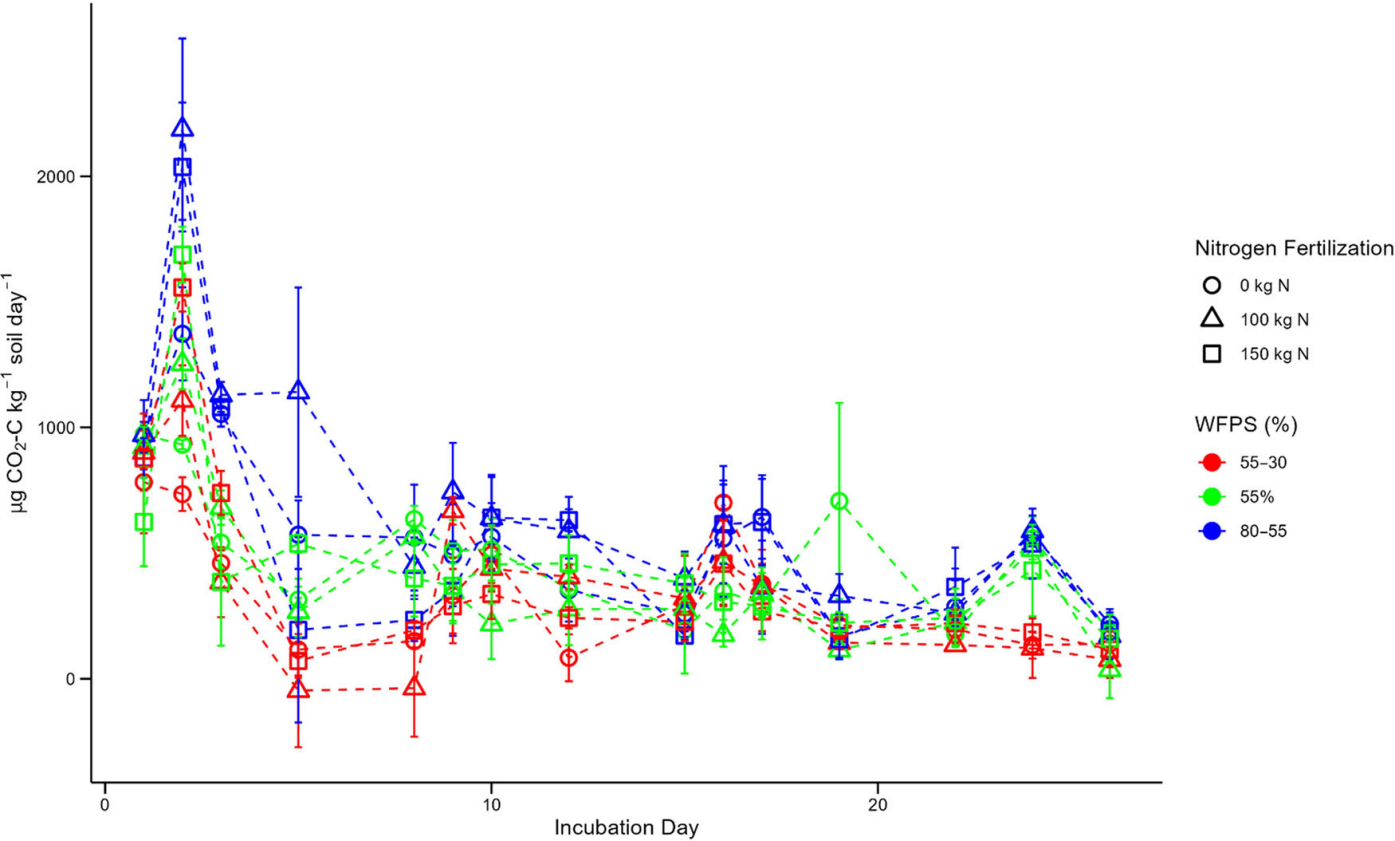
Daily CO_2_ production (µg CO_2_–C kg^−1^ soil day^−1^) during the incubation by treatment. Error bars represent ± 1 SE (standard error) for each treatment.

Daily CO_2_ production, under all treatments, progressively decreased as the incubation advanced (Figure 5). The highest rates of daily CO_2_ production, under all treatments, were recorded on day 2 of the incubation (Figure 5). However, the lowest daily rates of production overall were recorded on the final day (day 26) of the incubation (Figure 5). Daily CO_2_ production varied depending on the applied moisture regime, with production being the highest under wet‐fluctuating conditions and the lowest under dry‐fluctuating conditions (Figure 5). Minor increases in daily production were recorded in all treatments on day 9 and 16 of the incubation, with these being the first measurements taken after the pots were rewetted (to 55% and 80% WFPS, respectively; Figures 1 and 5).

Cumulative CO_2_ production, across the incubation, was also primarily determined by the experienced moisture regime and was highly significant (*p* < 0.001), whereas N fertilization rate was nonsignificant across the treatments (*p* > 0.05) (Table 2). Average cumulative CO_2_ production from all of the wet‐fluctuating pots, irrespective of N fertilization, was significantly higher than all of the dry‐fluctuating and constant‐baseline moisture pots (*p* < 0.01) (Table 2). Between the dry‐fluctuating and constant‐baseline moisture treatments, no significant differences were identified in cumulative CO_2_ production (*p* > 0.05) (Table 2).

## 4 DISCUSSION

### 4.1 N_2_O production

This study provides novel insights into the temporal and moisture‐dependent interactions between N fertilization and N_2_O emissions. While the control of N fertilization rate on N_2_O production and soil NO₃⁻ concentrations is well established (Bouwman et al., 2002; Lu et al., 2021; Pan et al., 2022), our results show that N fertilization rate significantly influences cumulative N_2_O production only under wet‐fluctuating conditions, highlighting the dependency of N_2_O emissions on soil moisture (Table 2, Figure 4). This temporal limitation of N fertilization effects on N_2_O production in drier conditions demonstrates that N fertilization's influence on emissions is not constant but varies with moisture availability.

While SOM‐N was the dominant source of N_2_O across all treatments, we observed that the contribution from urea increased significantly with higher WFPS and N fertilization rates (Table 2). This aligns with previous research highlighting the role of moderate moisture in facilitating nitrification, where ideal WFPS (55%–80%) supports the transformation of applied urea‐N to NO₃⁻ and subsequent N_2_O production via denitrification (Bateman & Baggs, 2005; Linn & Doran, 1984; Stevens et al., 1997). In the driest treatments, where nitrification is suppressed despite elevated NH₄⁺ concentrations, the lowest allocation of N_2_O derived from added urea was observed, underscoring the critical role of moisture in modulating N_2_O production from both fertilizer and organic matter sources (Table 2, Figure 4).

Additionally, the strongest PE were observed in the wet‐fluctuating treatments, where the remobilization and dissolution of SOM contributed to enhanced N_2_O production. This supports previous studies on positive PE under high soil moisture and SOM content (Schleusner et al., 2018; Thilakarathna & Hernandez‐Ramirez, 2021a). Increased moisture leads to greater SOM remobilization and a positive priming effect, enhancing N_2_O production. In contrast, under dry conditions, reduced SOM mineralization results in negative priming, suppressing N_2_O emissions (Kuzyakov et al., 2000; Thilakarathna & Hernandez‐Ramirez, 2021a).

Previous studies have reported the highest rates of N_2_O production at WFPS of 70%–80% (Liu et al., 2022; Qin et al., 2020). At higher WFPS, soils become increasingly anaerobic, reducing oxygen (O_2_) diffusion and enhancing denitrification, which increases N_2_O production along with conversion to N_2_ (Braker & Conrad, 2011; Cocco et al., 2018; Moldrup et al., 2000). Denitrification dominates N_2_O production when WFPS exceeds 60%–70%, while nitrification is the primary source when WFPS is below 60%–70% and above 20%–30% (Bateman & Baggs, 2005; Linn & Doran, 1984; Stevens et al., 1997). In relatively dry soils (<60% WFPS), nitrification is progressively inhibited as moisture decreases, with nitrifier activity sensitive to desiccation and substrate limitations (Gao et al., 2020; Hartmann et al., 2013; Linn & Doran, 1984). This pattern is consistent with the higher cumulative N_2_O production and soil NO₃⁻ concentrations observed under wet‐fluctuating treatments in our study (Table 2, Figure 4).

Although microbial gene expression or enzyme activity was not directly measured, the observed δ^15^N patterns likely reflect microbially mediated processes. Urease‐producing microbes facilitate urea hydrolysis to NH₄⁺, initiating N transformations (Byrnes & Freney, 1995; Krajewska, 2009). The moisture‐dependent increase in ^15^N‐derived N_2_O under wet conditions suggests enhanced denitrification, which is regulated by functional genes such as *nirK*, *nirS*, and *nosZ* (Jones et al., 2013; Philippot et al., 2007). Including microbial assays in future studies, such as urease activity or denitrification gene abundance, would strengthen isotopic source interpretations.

### 4.2 CO_2_ production

Peaks in CO_2_ emissions occurring on day 2 of the incubation can be attributed to the recent establishment of the experimental treatments following the measurements on day 1 (Figure 5). ^15^N‐labeled urea was incorporated into the corresponding microcosms following the day 1 measurements, while the wet‐fluctuating treatments saw their WFPS increase from 55% to 80% WFPS (Figure 1). These input disturbances could likely have increased soil microbial activity due to the rapid shift in moisture, N availability, and increased exposure of soil organic carbon, accelerating respiration rates and resultant CO_2_ production (Feng et al., 2013; Orchard & Cook, 1983). This is further supported by the observation that the unfertilized and dry‐fluctuating pots exhibited the lowest CO_2_ production on day 6 (Figure 5).

The highly significant and constant control of soil moisture on cumulative and daily CO_2_ production is well expected and documented within the literature. Prior studies have reported maximum CO_2_ production from soil at WFPS of 60% (W. Hassan et al., 2014; Linn & Doran, 1984), with decreases observed at higher and lower soil moistures. Under low soil moisture (<60% WFPS), CO_2_ production from respiration becomes limited due to a decreased supply of soluble substrates (Fairbairn et al., 2023; Schimel, 2018; Schjønning et al., 2003). Contrastingly, under high soil moisture (>60% WFPS), CO_2_ production begins to be limited by decreased soil aeration, as a result of the slower diffusion of O_2_ in saturated soil which causes aerobic respiration to be reduced (Fairbairn et al., 2023; Linn & Doran, 1984; Moldrup et al., 2000). However, these relationships are variable across soil conditions and textures, with Tang et al. (2016) and Zhang et al. (2020) both notably recording increases in CO_2_ production at 100% WFPS compared to 60% WFPS. As both Tang et al. (2016) and Zhang et al. (2020) suggested, the observation of the greatest CO_2_ production at 80% WFPS (Figures 1 and 5) can be attributed to the limited soil depths within the incubation pots (i.e., 5 cm), thus removing the limitation of O_2_ availability on aerobic respiration at relatively high soil moistures.

CO₂ emissions were interpreted as microbial respiration, but the study did not distinguish between carbon sources or microbial pathways. Future work using ^13^C‐labeled substrates or dual ^13^C–^15^;N isotopic approaches could help identify whether carbon dioxide production is derived from fertilizer inputs or SOM, and better link carbon and nitrogen cycling under varying moisture conditions (Daly & Hernandez‐Ramirez, 2020; Werth & Kuzyakov, 2010).

### 4.3 The impact of rewetting events on greenhouse gas production

Rewetting events serve as an additional control on both N_2_O and CO_2_ production, which leads to short‐term increases in production. Both daily N_2_O and CO_2_ production rates exhibited responses to rewetting events, specifically on day 9 and 16 of the incubation (Figures 2 and 5), these being the first measurements after moisture contents in the soil microcosms were restored to their upper limits (Figure 1). The recorded increases in daily N_2_O and CO_2_ production occurred over a single day, emphasizing that rewetting events exert a separate and distinct control on production from soil moisture. This temporality of increased N_2_O and CO_2_ production following rewetting was similarly reported by Harrison‐Kirk et al. (2013). Additionally, Harrison‐Kirk et al. (2013) also observed that the increase in CO_2_ production after rewetting was significantly lower than the increase in N_2_O production, which was attributed to a reduction in carbon mineralization during the drying phases.

The transient nature of the observed increases in production can be attributed to the rapid depletion of substrates following rewetting, which had initially risen as SOM was exposed by the disruption of soil aggregates during the rewetting events (Birch, 1958; Jin et al., 2023). Resultantly, while multiple rewetting events can occur across a growing season, the greatest increases in greenhouse gas production can be expected when there are extended gaps between rewetting events. These expectations are supported by the observed effects of rewetting on N_2_O priming production. Following the first rewetting event (day 9), N_2_O priming production peaked, with the wet‐fluctuating treatments being specifically elevated (Figure 3). These observations can again be explained by the greater SOM exposure due to the soil aggregate disruption caused by rewetting events (Birch, 1958; Jin et al., 2023). Rewetting events therefore both increase soil moisture and SOM availability, both of which are associated with positive priming events (Schleusner et al., 2018; Thilakarathna & Hernandez‐Ramirez, 2021a). Additionally, the decrease in PE observed following the second rewetting event (incubation day 16) can be attributed to a decrease in readily available SOM at this later stage of the incubation after consecutive wet‐drying cycles.

While short‐term PE were inferred from increased greenhouse gas production following rewetting events, the magnitude of priming was relatively modest and mechanistic interpretation remains limited. Microbial community composition or extracellular enzyme activities were not assessed, both of which are known to mediate priming responses through altered SOM decomposition and N cycling dynamics (Blagodatskaya & Kuzyakov, 2008; Fontaine et al., 2004). Future work incorporating microbial functional data (e.g., gene expression or enzyme assays) could clarify the biological drivers of priming and help link microbial responses to changes in greenhouse gas production more directly.

## 5 CONCLUSION

This study highlights the complex interplay between fluctuating soil moisture and N fertilization in influencing both the total amount of N_2_O produced and the relative contributions of different N sources, such as added urea and SOM. Specifically, soil moisture was found to modulate the balance of N_2_O production, with higher moisture levels increasing the contribution of urea to emissions, even though SOM remained the dominant source. This adds a new layer of complexity to our understanding of greenhouse gas production in fertilized agricultural systems. The findings underscore the dynamic and moisture‐dependent nature of the fertilization–N_2_O relationship, emphasizing the critical need for effective moisture management in efforts to mitigate N_2_O emissions. In addition, the results demonstrate that fluctuating moisture plays a significant role in controlling soil N_2_O and CO_2_ production, with wetter conditions consistently leading to higher emissions compared to drier regimes. The influence of N fertilization on N_2_O production was also evident, particularly in urea‐amended soils. However, the effect of fertilization was temporally limited and strongly influenced by soil moisture content, with a stronger initial impact that diminished over time as nitrification progressed. While the roles of N fertilization and soil moisture on greenhouse gas emissions are well established, this study further emphasizes how their interaction can shape emission patterns in agricultural systems.

## AUTHOR CONTRIBUTIONS

**Matt Ball**: Data curation; formal analysis; writing—original draft. **Guillermo Hernandez‐Ramirez**: Funding acquisition; methodology; resources; writing—review and editing.

## CONFLICT OF INTEREST STATEMENT

The authors declare no conflicts of interest.
